# Supplementary material for: Projections of faster onset and slower decay of El Niño in the 21st century
Source: Nat Commun. 2022 Apr 8;13:1915. doi: 10.1038/s41467-022-29519-7 (PMC8993828; doi:10.1038/s41467-022-29519-7)
Supplement: Supplementary file 1 — Supplementary Information [file 41467_2022_29519_MOESM1_ESM.pdf]

Supplemental Information

Projections of Faster Onset and Slower Decay of El Niño in the 21st Century

Hosmay Lopez[1], Sang-Ki Lee[1], Dongmin Kim[2,1], Andrew T. Wittenberg[3], and Sang-Wook Yeh[4]

[1]Atlantic Oceanographic and Meteorological Laboratory, NOAA, Miami, FL

[2]Cooperative Institute for Marine and Atmospheric Studies, University of Miami, Miami, FL

[3]Geophysical Fluid Dynamics Laboratory, NOAA, Princeton, NJ

[4]Department of Marine Science and Convergent Technology, Hanyang University, Ansan, Korea

Included materials:

Supplementary Tables 1, 2, 3, and 4

Supplementary Figures 1, 2, 3, 4, 5, 6, 7, 8, 9, 10, 11, 12, 13, 14, 15, and 16

Supplementary Note 1: Climate models analyzed in this study

Supplementary Table 1. Climate models used in the study. The asterisk for CESM1 indicates the CESM-LENS simulation, using 30 ensemble members for the historical and RCP8.5 emission scenarios from CMIP5. All other models were analyzed using a single ensemble member each for the historical and SSP585 emission scenarios from CMIP6.

Model Name	Institution, Country	Ensemble	References (historical, SSP585)
ACCESS-CM2	CSIRO, Australia	r1i1p1f1	1,2
ACCESS-ESM1.5	CSIRO, Australia	r1i1p1f1	3.4
CAMS-CSM1-0	CAMS, China	r1i1p1f1	5,6
CanESM5	CCCma, Canada	r1i1p1f1	7.8
CESM1-LENS*	NCAR, USA	r1i1p1f1	9
CESM2	NCAR, USA	r1i1p1f1	10,11
CESM2-WACCM	NCAR, USA	r1i1p1f1	12,13
EC-Earth3	European Consortium	r1i1p1f1	14,15
EC-Earth3-Veg	European Consortium	r1i1p1f1	16,17
GFDL-CM4	NOAA/GFDL, USA	r1i1p1f1	18,19
GFDL-ESM4	NOAA/GFDL, USA	r1i1p1f1	20,21
HadGM3-GC31-LL	MOHC, UK	r1i1p1f3	22,23
MIROC6	JAMSTEC, Japan	r1i1p1f1	24,25
MIROC-ES2L	JAMSTEC, Japan	r1i1p1f2	26,27
MPI-ESM1.2-LR	MPI-M, Germany	r1i1p1f1	28,29
MRI-ESM2.0	MRI, Japan	r1i1p1f1	30,31
NorESM2-LM	NCC, Norway	r1i1p1f1	32,33

Supplementary References for CMIP6 models and CESM-LENS

1. Dix, M. et al. CSIRO-ARCCSS ACCESS-CM2 model output prepared for CMIP6 CMIP Historical Version 2020201 (Earth System Grid Federation, 2019); https://doi.org/10.22033/ESGF/CMIP6.4271
2. Dix, M. et al. CSIRO-ARCCSS ACCESS-CM2 model output prepared for CMIP6 ScenarioMIP ssp585 Version 20210201 (Earth System Grid Federation, 2019); https://doi.org/10.22033/ESGF/CMIP6.4332
3. Ziehn, T. et al. CSIRO ACCESS-ESM1.5 Model Output Prepared for CMIP6 CMIP Historical Version 20210201 (Earth System Grid Federation, 2019); https://doi.org/10.22033/ESGF/CMIP6.4272
4. Ziehn, T. et al. CSIRO ACCESS-ESM1.5 Model Output Prepared for CMIP6 ScenarioMIP ssp585 Version 20210201 (Earth System Grid Federation, 2019); https://doi.org/10.22033/ESGF/CMIP6.4333
5. Rong, X. et al. CAMS CAMS-CSM1.0 Model Output Prepared for CMIP6 CMIP Historical Version 20210201 (Earth System Grid Federation, 2019); https://doi.org/10.22033/ESGF/CMIP6.9754

6. Rong, X. et al. CAMS CAMS-CSM1.0 Model Output Prepared for CMIP6 ScenarioMIP ssp585 Version 20210201 (Earth System Grid Federation, 2019); https://doi.org/10.22033/ESGF/CMIP6.11052
7. Swart, N. C et al. *CCCma CanESM5 model output prepared for CMIP6* CMIP Historical Version *2021020. (* Earth System Grid Federation, 2019). https://doi.org/10.22033/ESGF/CMIP6.1317
8. Swart, N. C et al. *CCCma CanESM5 model output prepared for CMIP6* ScenarioMIP ssp585 Version *2021020. (* Earth System Grid Federation, 2019). https://doi.org/10.22033/ESGF/CMIP6.1317
9. Kay, J. Deser, C. Phillips, A. Mai, A. Hannay, C. Strand, G. Arblaster, The Community Earth System Model (CESM) Large Ensemble Project: A community resource for studying climate change in the presence of internal climate variability. *Bull. Am. Meteorol. Soc.* **96,** 1333–1349 (2015). https://doi.org/10.1175/BAMS-D-13-00255.1
10. Danabasoglu, G. NCAR CESM2 Model Output Prepared for CMIP6 CMIP Historical Version 20210201 (Earth System Grid Federation, 2019); https://doi.org/10.22033/ESGF/CMIP6.7627
11. Danabasoglu, G. NCAR CESM2 Model Output Prepared for CMIP6 ScenarioMIP ssp585 Version 20210201 (Earth System Grid Federation, 2019); https://doi.org/10.22033/ESGF/CMIP6.7768
12. Danabasoglu, G. NCAR CESM2-WACCM Model Output Prepared for CMIP6 CMIP Historical Version 20210201 (Earth System Grid Federation, 2019); https://doi.org/10.22033/ESGF/CMIP6.10071
13. Danabasoglu, G. NCAR CESM2-WACCM Model Output Prepared for CMIP6 ScenarioMIP ssp585 Version 20210201 (Earth System Grid Federation, 2019); https://doi.org/10.22033/ESGF/CMIP6.10115
14. EC-Earth Consortium (EC-Earth). EC-Earth-Consortium EC-Earth3 Model Output Prepared for CMIP6 CMIP Historical Version 20210201 (Earth System Grid Federation, 2019); https://doi.org/10.22033/ESGF/CMIP6.4700
15. EC-Earth Consortium (EC-Earth). EC-Earth-Consortium EC-Earth3 Model Output Prepared for CMIP6 ScenarioMIP ssp585 Version 20210201 (Earth System Grid Federation, 2019); https://doi.org/10.22033/ESGF/CMIP6.4912
16. EC-Earth Consortium (EC-Earth). EC-Earth-Consortium EC-Earth3-Veg Model Output Prepared for CMIP6 CMIP Historical Version 20210201 (Earth System Grid Federation, 2019); https://doi.org/10.22033/ESGF/CMIP6.4706
17. EC-Earth Consortium (EC-Earth). EC-Earth-Consortium EC-Earth3-Veg Model Output Prepared for CMIP6 ScenarioMIP ssp585 Version 20210201 (Earth System Grid Federation, 2019); https://doi.org/10.22033/ESGF/CMIP6.4914
18. Guo, H. et al. NOAA-GFDL GFDL-CM4 Model Output Prepared for CMIP6 CMIP Historical Version 20210201 (Earth System Grid Federation, 2018); https://doi.org/10.22033/ESGF/CMIP6.8594
19. Guo, H. et al. NOAA-GFDL GFDL-CM4 Model Output Prepared for CMIP6 ScenarioMIP ssp585 Version 20210201 (Earth System Grid Federation, 2018); https://doi.org/10.22033/ESGF/CMIP6.9268
20. Krasting, J. P. et al. NOAA-GFDL GFDL-ESM4 Model Output Prepared for CMIP6 CMIP Historical Version 20210201 (Earth System Grid Federation, 2018); https://doi.org/10.22033/ESGF/CMIP6.8597 114.

21. Krasting, J. P. et al. NOAA-GFDL GFDL-ESM4 Model Output Prepared for CMIP6 ScenarioMIP ssp585 Version 20210201 (Earth System Grid Federation, 2018); https://doi.org/10.22033/ESGF/CMIP6.8706
22. Ridley, J. et al. MOHC HadGEM3-GC31-LL Model Output Prepared for CMIP6 CMIP Historical Version 20210201 (Earth System Grid Federation, 2019); https://doi.org/10.22033/ESGF/CMIP6.6109
23. Good, P. et al. MOHC HadGEM3-GC31-LL Model Output Prepared for CMIP6 ScenarioMIP ssp585 Version 20210201 (Earth System Grid Federation, 2020); https://doi.org/10.22033/ESGF/CMIP6.10901
24. Tatebe, H. & Watanabe, M. MIROC MIROC6 Model Output Prepared for CMIP6 CMIP Historical Version 20210201 (Earth System Grid Federation, 2019); https://doi.org/10.22033/ESGF/CMIP6.5603
25. Shiogama, H., Abe, M. & Tatebe, H. MIROC MIROC6 Model Output Prepared for CMIP6 ScenarioMIP ssp585 Version 20210201 (Earth System Grid Federation, 2019); https://doi.org/10.22033/ESGF/CMIP6.5771
26. Hajima, T. et al. MIROC MIROC-ES2L Model Output Prepared for CMIP6 CMIP Historical Version 20210201 (Earth System Grid Federation, 2019); https://doi.org/10.22033/ESGF/CMIP6.5602
27. Tachiiri K. et al. MIROC MIROC-ES2L Model Output Prepared for CMIP6 ScenarioMIP ssp585 Version 20210201 (Earth System Grid Federation, 2019); https://doi.org/10.22033/ESGF/CMIP6.5770
28. Wieners, K.-H. et al. MPI-M MPI-ESM1.2-LR Model Output Prepared for CMIP6 CMIP Historical Version 20210201 (Earth System Grid Federation, 2019); https://doi.org/10.22033/ESGF/CMIP6.6595
29. Wieners, K.-H. et al. MPI-M MPI-ESM1.2-LR Model Output Prepared for CMIP6 ScenarioMIP ssp585 Version 20210201 (Earth System Grid Federation, 2019); https://doi.org/10.22033/ESGF/CMIP6.6705
30. Yukimoto, S. et al. MRI MRI-ESM2.0 Model Output Prepared for CMIP6 CMIP Historical Version 20210201 (Earth System Grid Federation, 2019); https://doi.org/10.22033/ESGF/CMIP6.6842
31. Yukimoto, S. et al. MRI MRI-ESM2.0 Model Output Prepared for CMIP6 ScenarioMIP ssp585 Version 20210201 (Earth System Grid Federation, 2019); https://doi.org/10.22033/ESGF/CMIP6.6929
32. Seland, Ø. et al. NCC NorESM2-LM Model Output Prepared for CMIP6 CMIP Historical Version 20210201 (Earth System Grid Federation, 2019); https://doi.org/10.22033/ESGF/CMIP6.8036
33. Seland, Ø. et al. NCC NorESM2-LM Model Output Prepared for CMIP6 ScenarioMIP ssp585 Version 20210201 (Earth System Grid Federation, 2019); https://doi.org/10.22033/ESGF/CMIP6.8319

Supplementary Note 2: Changes in El Niño statistics based on fixed area-averaged indices

We followed Fang and Yu 2020 by defining onset, duration, and demise months based on SSTA exceeding 0.5°C. We have done so for different ENSO indices given the spatio-temporal diversity changes (see tables 2 and 3 below):

Supplementary Table 2. Composite of basic statistics for El Niño events in the 20C and 21C simulations from the CESM-LENS using the RCP8.5 projection scenario, for three common SSTA indices (Niño3, Niño3.4 and Niño4). The amplitude is measured as the maximum SSTA reached, start day is measured as the first day when the SSTA>0.5°C, peak month is the month when the maximum amplitude is reached for all ensembles, end day is the last day of SSTA>0.5°C, growth (decay) rate is measured as the rate of change of SSTA from the start (peak) day to the peak (end) day. Entries in bold fonts depict significant changes between 20C and 21C determined from the ensemble spread based on a bootstrapping technique at a 95% confidence level (see Methods). Total events analyzed were 350 for the 20C, and 419 for the 21C.

SSTA Index	Case	Amplitude °C	Start Day range	Peak month	End Day range	Growth Rate °C/month	Decay Rate °C/month
Nino3	20C	1.33 ± 0.07	**08 Apr – 21 Apr**	Dec	**21 Feb – 30 Mar**	0.09 ± 0.01	**-0.30 ± 0.04**
	21C	1.37 ± 0.06	**25 Apr – 03 May**	Dec	**09 Apr – 26 Apr**	0.12 ± 0.05	**-0.16 ± 0.02**
Nino3.4	20C	**1.92 ± 0.06**	18 Apr – 04 May	**Jan**	**16 May – 26Jun**	**0.15 ± 0.01**	-0.27 ± 0.03
	21C	**2.09 ± 0.06**	31 Mar – 24 Apr	**Dec**	**01 Apr - 13May**	**0.22 ± 0.03**	-0.26 ± 0.05
Nino4	20C	1.65 ± 0.06	**01 Feb – 07 Mar**	**Jan**	09 May – 14 Jun	**0.11 ± 0.01**	**-0.19 ± 0.01**
	21C	1.77 ± 0.07	**08 Mar – 12 May**	**Dec**	06 Apr – 24 May	**0.16 ± 0.03**	**-0.26 ± 0.02**

Supplementary Table 3. Same as Supplementary Table 2, but for the CMIP6 model simulations, using the SSP585 projection scenario for 21C. Total events analyzed were 199 for the 20C, and 234 for the 21C.

SSTA Index	Case	Amplitude °C	Start Day range	Peak month	End Day range	Growth Rate °C/month	Decay Rate °C/month
Nino3	20C	**1.21 ± 0.04**	08 May – 14 May	Dec	**1 Apr – 11 May**	0.08 ± 0.01	**-0.15 ± 0.02**
	21C	**1.36 ± 0.04**	11 May – 18 May	Jan	**16 May – 28 Jun**	0.09 ± 0.02	**-0.11 ± 0.01**
Nino3.4	20C	**1.55 ± 0.03**	17 May – 29 May	**Jan**	07 May – 16 May	**0.14 ± 0.01**	**-0.20 ± 0.01**
	21C	**1.86 ± 0.05**	06 May – 17 May	**Dec**	04 Apr – 19 May	**0.19 ± 0.02**	**-0.25 ± 0.02**
Nino4	20C	**1.10 ± 0.03**	**14 Jun – 27 Jun**	**Jan**	**09 May – 30 Jun**	0.09 ± 0.01	**-0.11 ± 0.01**
	21C	**1.23 ± 0.05**	**23 May – 12 Jun**	**Dec**	**03 Mar – 10 Apr**	0.10 ± 0.02	**-0.15 ± 0.02**

Fang, S. W., & Yu, J. Y. (2020). Contrasting transition complexity between El Niño and La Niña: Observations and CMIP5/6 models. Geophysical Research Letters, 47(16), e2020GL088926.

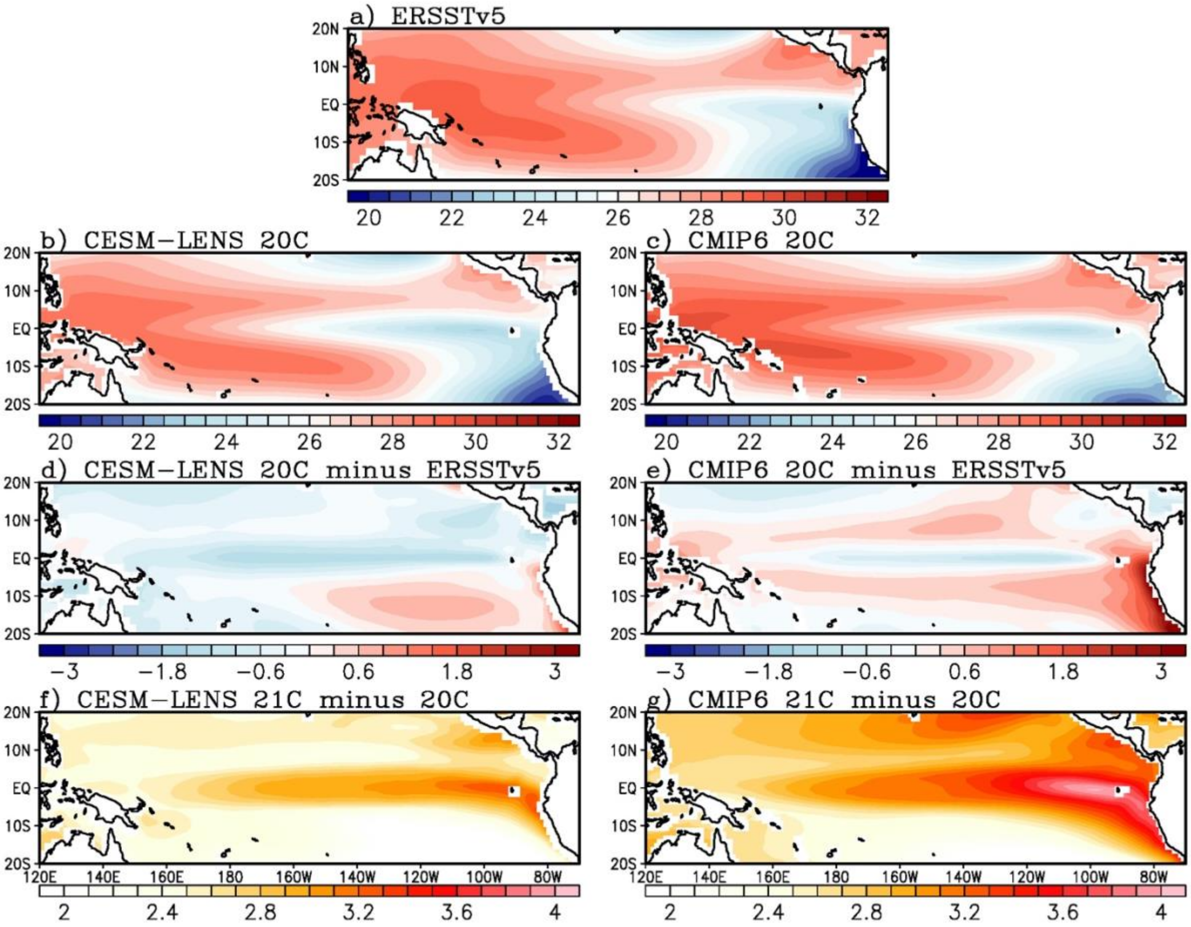

Supplementary Figure 1. Tropical Pacific mean sea surface temperature (SST) for the late 20C (1951-2000), from a) observational estimates from ERSSTv5, b) CESM-LENS simulation, and c) CMIP6 simulation. Panels d) and e) show the SST biases for CESM-LENS and CMIP6 relative to ERSSTv5. Panels f) and g) show the projected time-mean changes in the late 21C (2051-2100) relative to the late 20C.

Supplementary Note 3: Changes in the likelihood of El Niño occurrence

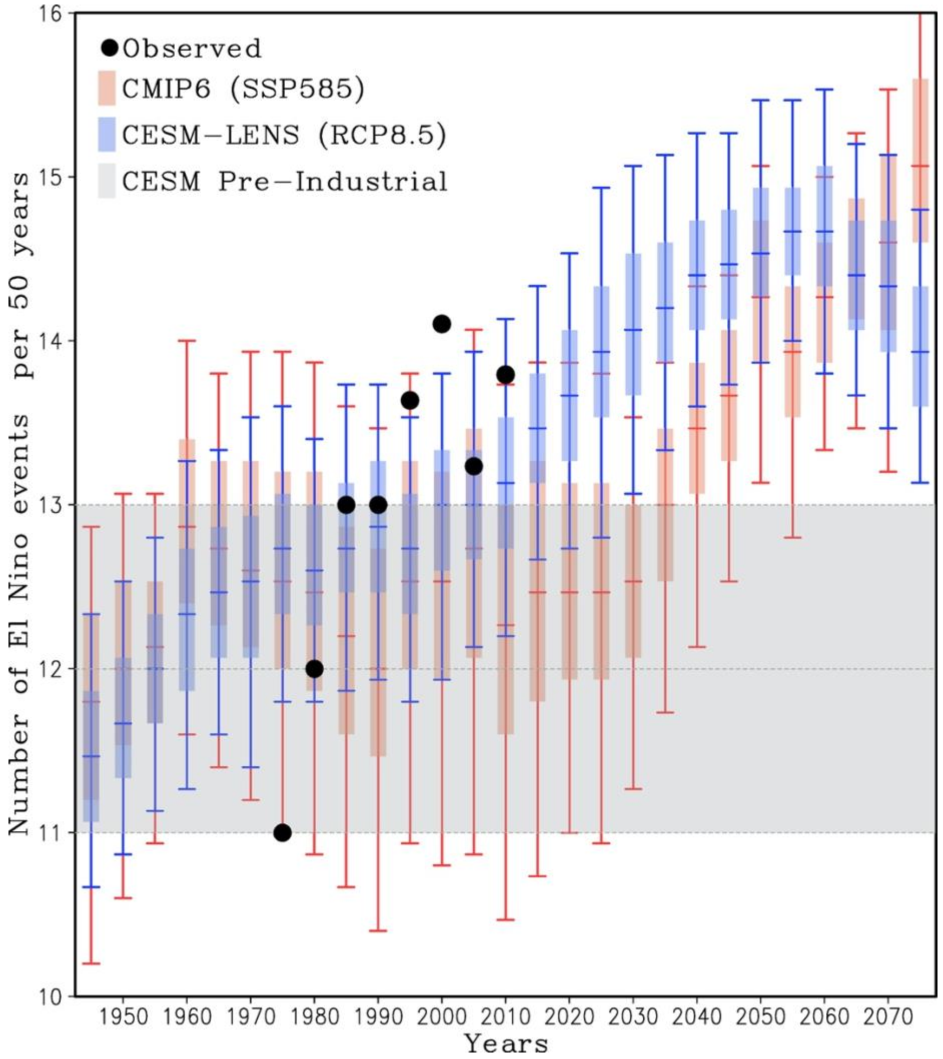

Supplementary Figure 2. Temporal evolution of a 50-year running averaged number of El Niño events from observations (black dots, ERSSTv5), CMIP6 (red), and CESM-LENS simulations (blue). The boxes denote the interquartile range, and the whiskers denote 5th-95th percentile range, of event counts estimated by randomly selecting 8 out of 16 (15 out of the 30) ensemble members 1000 times and repeating the event count for CMIP6 (CESM-LENS) respectively. The gray interval denotes the natural variability range, computed from the interquartile range from a 1100-year pre-industrial simulation of CESM by randomly selecting 50-year periods for the event count. The year labels on the abscissa correspond to the central year of the 50-year window. For example, the year 2070 indicates the period spanning 2046-2095. The 21C projections are from the SSP585 (RCP8.5) scenario from CMIP6 (CESM-LENS) respectively.

Supplementary Note 4: Analysis of the spatio-temporal diversity of El Niño and its future projections.

As suggested in the title and verified by the major findings, ENSO diversity in the future is too complicated to assess based on fixed geographical indices, such as the typical EP and CP definitions. Also, the EP and CP distinction does not address the projected temporal changes.

Given this, we follow the definition of Lee et al. 2018 for the spatio-temporal diversity of El Niño and repeated the analysis for the 20C and 21C separately for the CESM-LENS. In brief, we performed an empirical orthogonal function analysis of the spatio-temporal evolution of El Niño SSTA. The method is as follow:

For the 20C and 21C period, an SSTA is constructed as a function of longitude and time for each El Niño event e.g., SSTA (longitude, time, event), where the longitude-time domain is the same as the one used throughout the paper (i.e., Fig. 1). We then perform an EOF of the event covariance matrix after removing the ensemble mean (i.e., ensemble mean is the mean of all events as shown in Fig. 1) as follows:

$$EL\ Niño\ Diversity = EOF(X)$$

Where X is the covariance of $SSTA(lon, time, event) - SSTA_{ensemble\ mean}(lon, time)$

As in Lee et al. 2018, we only retained the leading two modes of spatio-temporal El Niño variability accounting for a combined 75.2% variance explained for the 20C and 75.9% variance explained for the 21C period.

El Niño diversity is then reconstructed as:

- $EL\ Niño\ (transitioning\ flavor) = SSTA_{ensemble\ mean}(lon, time) + EOF1$
- $EL\ Niño\ (resurgent\ flavor) = SSTA_{ensemble\ mean}(lon, time) - EOF1$
- $EL\ Niño\ (persitent\ flavor) = SSTA_{ensemble\ mean}(lon, time) + EOF2$
- $EL\ Niño\ (early\ terminating\ flavor) = SSTA_{ensemble\ mean}(lon, time) - EOF2$

Supplementary Figure 3 shows the spatio-temporal evolution of each El Niño flavor and the bi-variate distribution of the principal components (PC1 and PC2). Panels a, b, c, and d, are consistent with Lee et al. Note that the persistent (resurgent) event flavor is projected to increase by 47% (38%), which is significant at a 95% confidence level based on a Monte Carlo random sampling technique. The transitioning and early-terminating flavors are projected to increase by 15% and 21% respectively. Recall that there is a total of 350 events in the 20C and 419 events in the 21C, for an increase of 19.7%. Therefore, any comparison of projected increase of a specific flavor should be relative the 19.7% projected increase of all events which also include the mixed flavors.

While the projected changes reported here occur in all types of El Niño, the persistent, and to a lesser degree, the resurgent types of El Niño dominate the reported increase of El Niño occurrence. This is consistent with the main result in the original paper which show an increase in the persistence of the events into the boreal spring and summer.

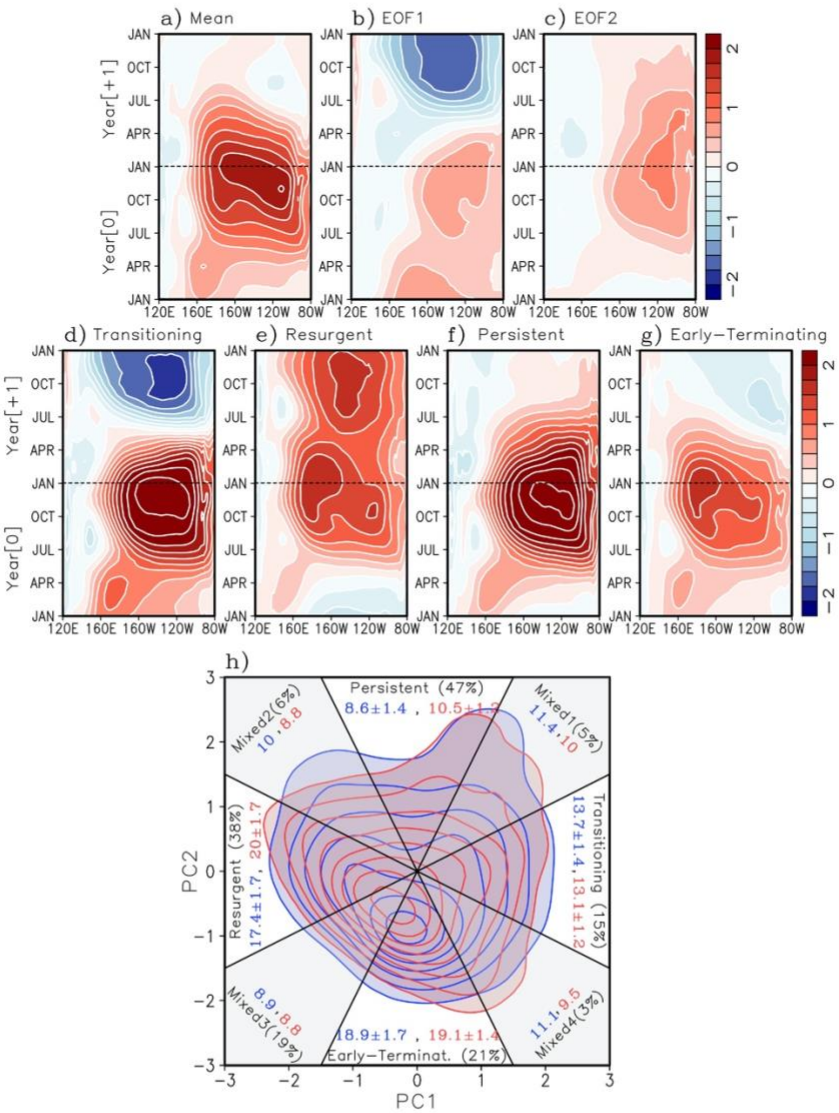

Supplementary Figure 3. Spatio-temporal El Niño diversity from CESM-LENS 20C expressed from the combination of the two leading empirical orthogonal functions. a) composite mean, b) EOF1, and c) EOF2 of the longitude-time evolution of SSTA. d) SSTA evolution of the transitioning El Niño flavor (e.g., composite mean + EOF1), e) resurgent flavor (e.g., composite mean - EOF1), f) persistent flavor (e.g., composite mean + EOF2), and g) early-terminating flavor (e.g., composite mean - EOF2). Panel h) shows the 20C (blue) and 21C (red) bi-variate distribution of the phase-space relationship between principial components (PC1 and PC2). The percentage values in parentheses indicate the projected percentage increase in the specific event flavor in the 21C relative to the 20C. The blue (red) values indicate the percentage of events in each category out of the total events for the 20C (21C).

Supplementary Note 5: Residual heat budget decomposition. Surface net heat fluxes and non-linear advection

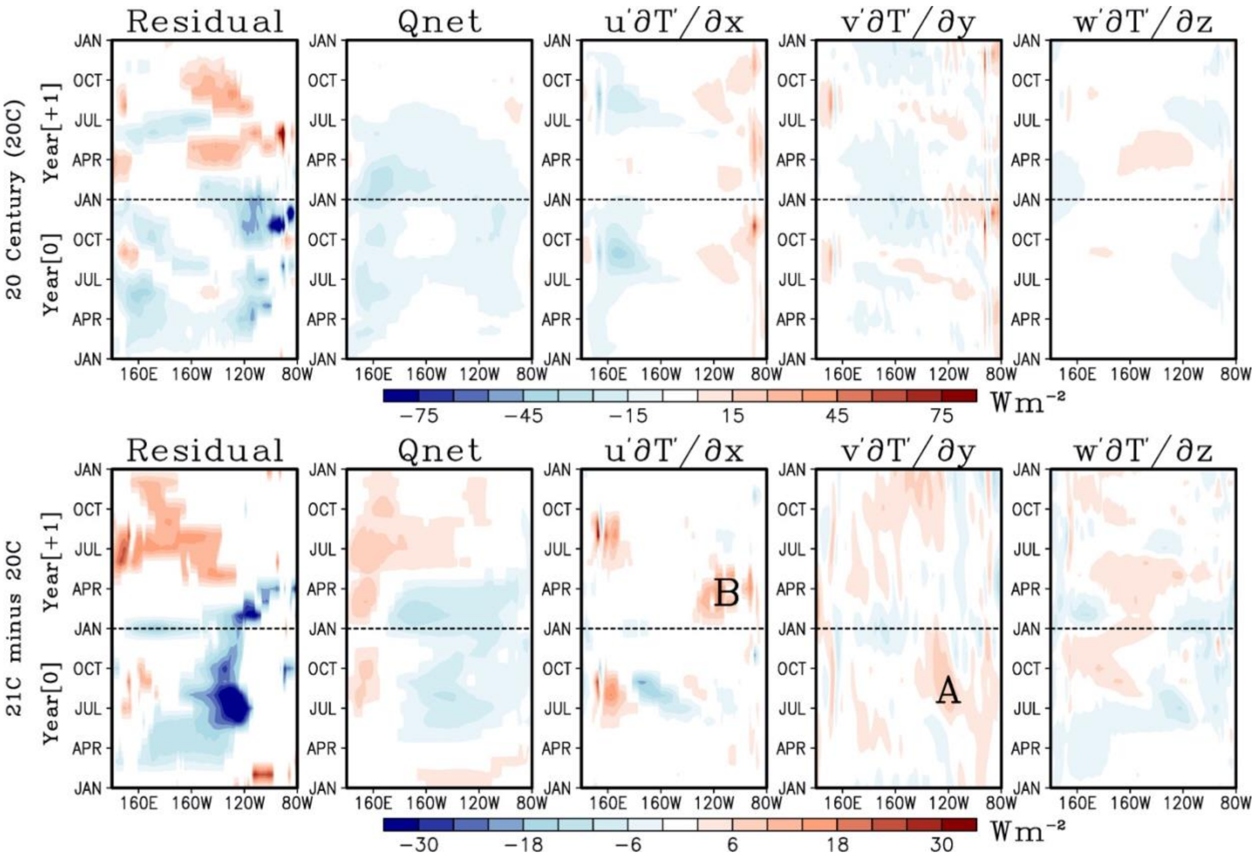

Supplementary Figure 4. Similar to Fig. 2 but showing the residual terms of the mixed layer heat budget analysis during El Niño events for CESM-LENS (*W m*^{2}). The top row shows the composites for the residual, air-sea net heat fluxes, and the three nonlinear advective terms (i.e., zonal, meridional, and vertical advection). Bottom row shows the projected future change in the composite, for the 21st minus the 20th Century (21C). All terms are computed assuming a constant 75 m mixed layer depth. Only showing anomalies that exceed the 95% confidence level based on a bootstrapping technique. See text for references to points A and B.

Supplementary Note 6: Relative contributions of mean state changes versus ENSO-induced anomaly changes in major feedback terms.

Supplementary Table 4. Relative contribution of changes in the mean climate (i.e., overbar) and changes in ENSO (i.e., prime) to the total feedback terms. The subscripts indicate whether the terms are evaluated in the 20C or 21C period.

	Thermocline Feedback	Zonal Advective Feedback	Ekman Feedback
Total changes	$\bar{w}_{21C}\frac{\partial T'_{21C}}{\partial z} - \bar{w}_{20C}\frac{\partial T'_{20C}}{\partial z}$	$u'_{21C}\frac{\partial \bar{T}_{21C}}{\partial x} - u'_{20C}\frac{\partial \bar{T}_{20C}}{\partial x}$	$w'_{21C}\frac{\partial \bar{T}_{21C}}{\partial z} - w'_{20C}\frac{\partial \bar{T}_{20C}}{\partial z}$
Changes in anomalies	$\bar{w}_{20C}\frac{\partial T'_{21C}}{\partial z} - \bar{w}_{20C}\frac{\partial T'_{20C}}{\partial z}$	$u'_{21C}\frac{\partial \bar{T}_{20C}}{\partial x} - u'_{20C}\frac{\partial \bar{T}_{20C}}{\partial x}$	$w'_{21C}\frac{\partial \bar{T}_{20C}}{\partial z} - w'_{20C}\frac{\partial \bar{T}_{20C}}{\partial z}$
Changes in the mean	$\bar{w}_{21C}\frac{\partial T'_{20C}}{\partial z} - \bar{w}_{20C}\frac{\partial T'_{20C}}{\partial z}$	$u'_{20C}\frac{\partial \bar{T}_{21C}}{\partial x} - u'_{20C}\frac{\partial \bar{T}_{20C}}{\partial x}$	$w'_{20C}\frac{\partial \bar{T}_{21C}}{\partial z} - w'_{20C}\frac{\partial \bar{T}_{20C}}{\partial z}$

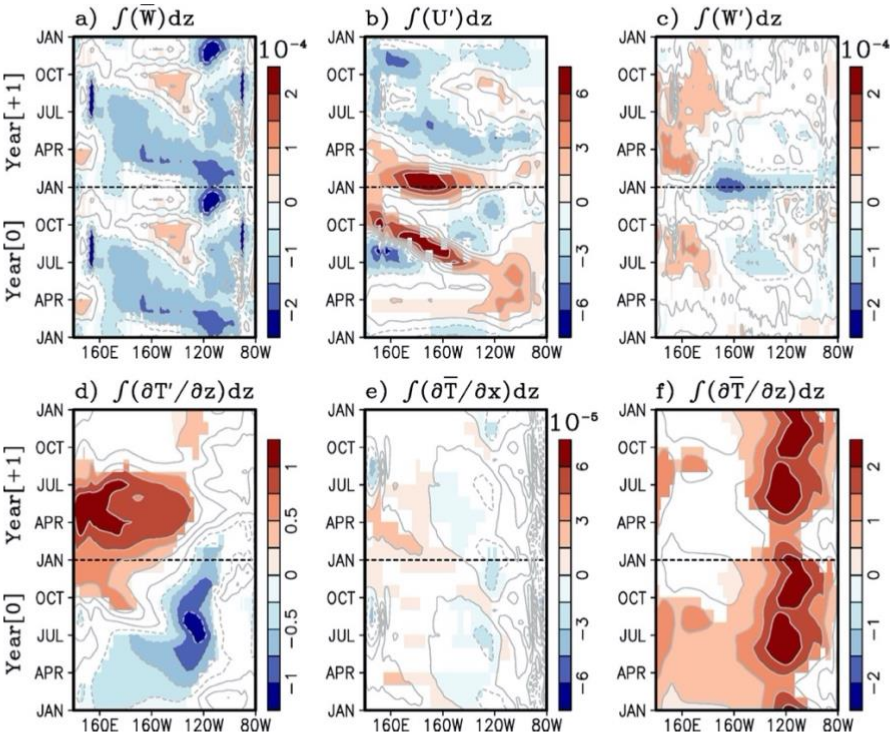

Supplementary Figure 5. Composite difference between the 21st minus the 20th Century changes in temperature and velocity contributions to the thermocline (left-column), zonal advective (middle-column), and Ekman (right-column) feedback components during El Niño events from CESM-LENS. The x-axis represents longitude across the Pacific Ocean and y-axis represents time from January of the onset year (Year 0) to December of the decay year (Year 1). The composite is meridionally averaged from 5°S to 5°N.

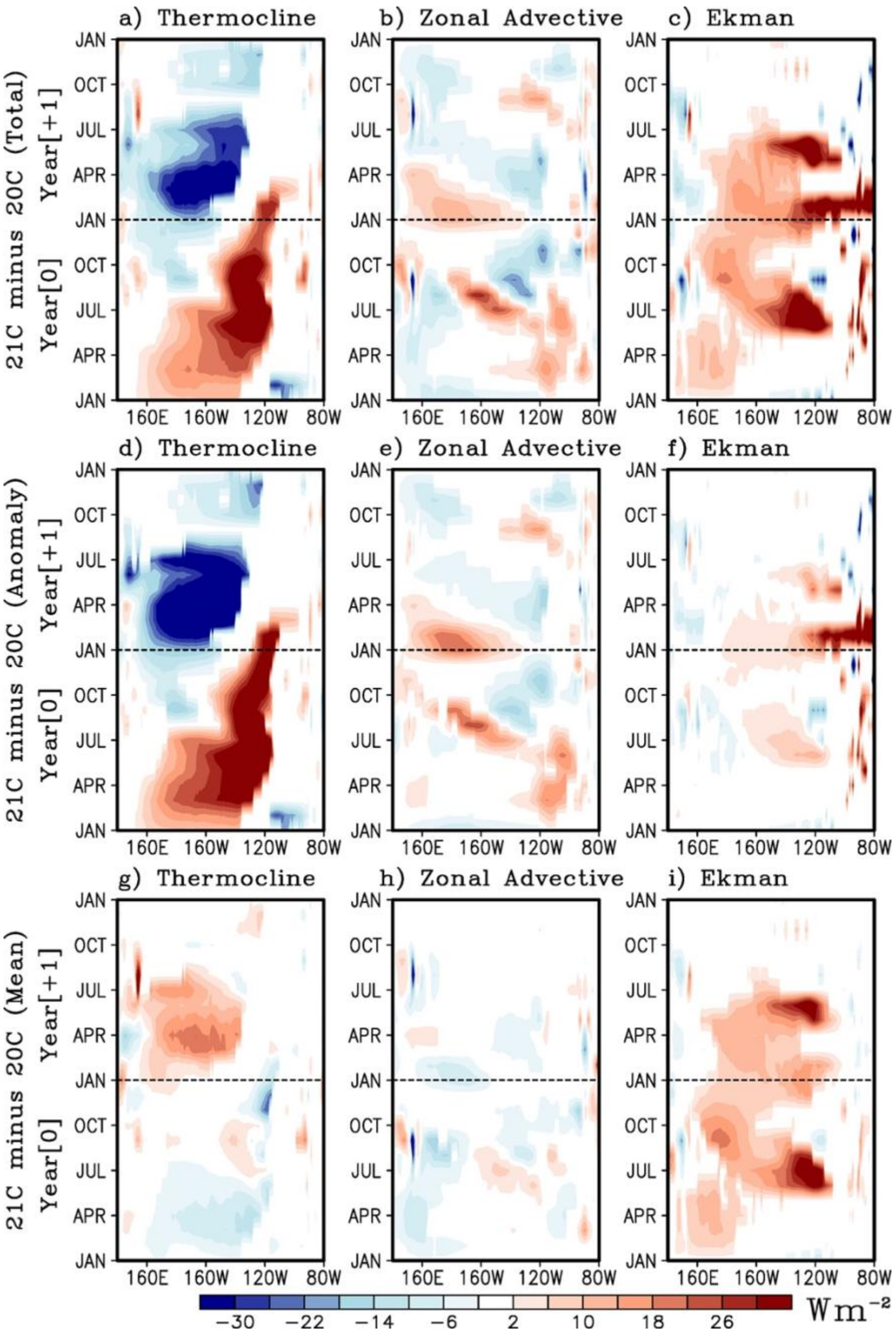

Supplementary Figure 6. Similar to supplementary Fig. 5, but for the composite difference between the 21st minus the 20th Century of a) thermocline, b) zonal advective, and c) Ekman feedbacks. Similarly, d), e) and f) show the contribution of projected changes in anomalies to each feedback while keeping the mean state to that of the 20th Century mean state (i.e., overbar terms in eq. 1 are from the 20th Century period while primes are for each respective Century). Similarly, g), h), and i) correspond to the contribution of projected mean state changes to each feedback if computed from just 20th Century anomalies (e.g., prime terms in eq. 1 are from 20th Century while the overbar terms are for their respective century). Refer to Table S4 for details on calculation.

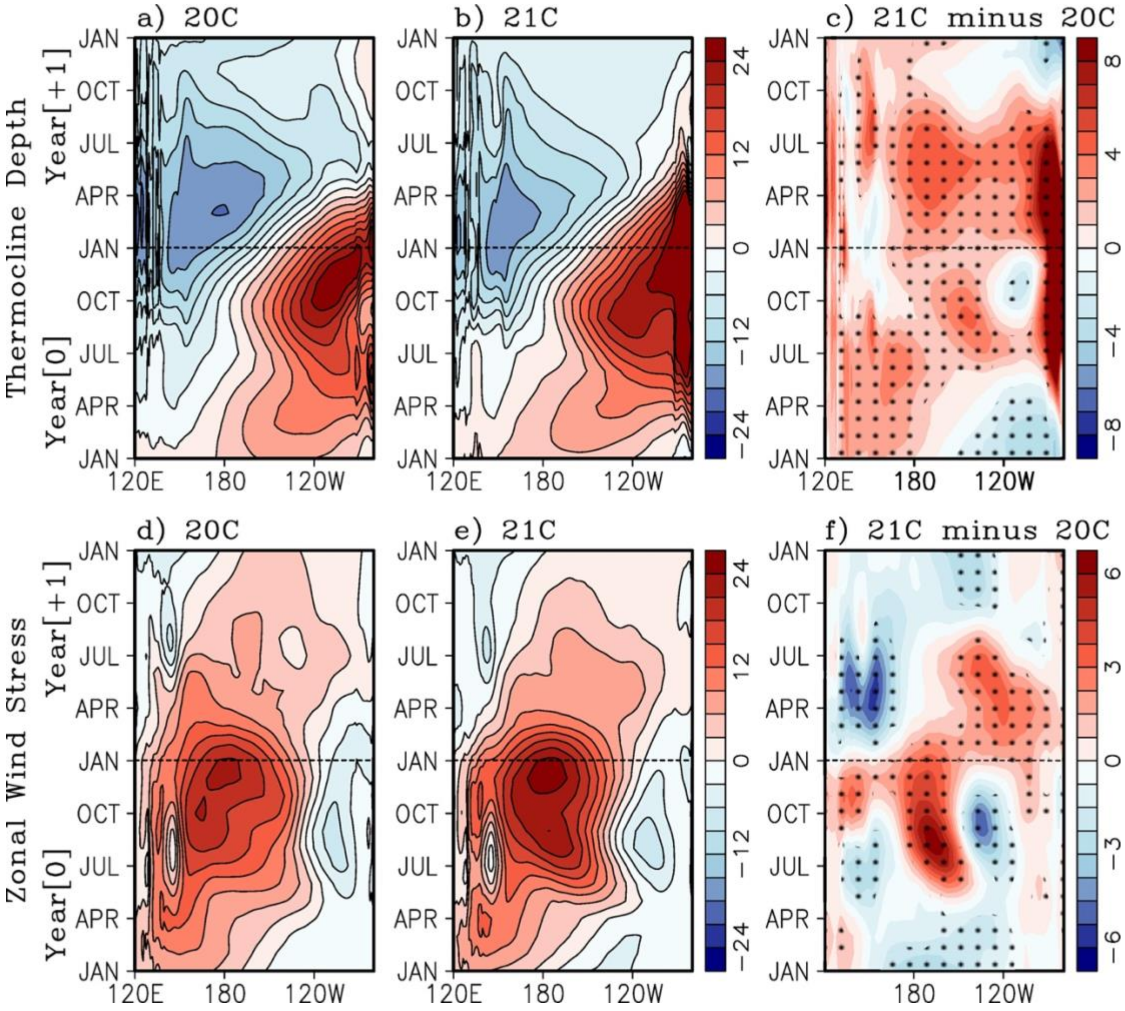

Supplementary Figure 7. Similar to supplementary Fig. 5, but for (top) composite of monthly thermocline depth anomaly [m] for El Niño events for a) the 20th Century, b) 21st Century, and c) the 21st minus 20th Century difference. Similarly, d), e), and f) show the composite monthly zonal wind stress anomalies [10^{-2} N m^{-2}]. The x-axis represents longitude across the Pacific Ocean and y-axis represents time from January of the onset year (Year 0) to December of the decay year (Year 1). The composite is meridionally averaged from 5°S to 5°N. Hatching on panels c) and f) indicate statistical significance at the 95% confidence level using a bootstrapping technique (see Methods).

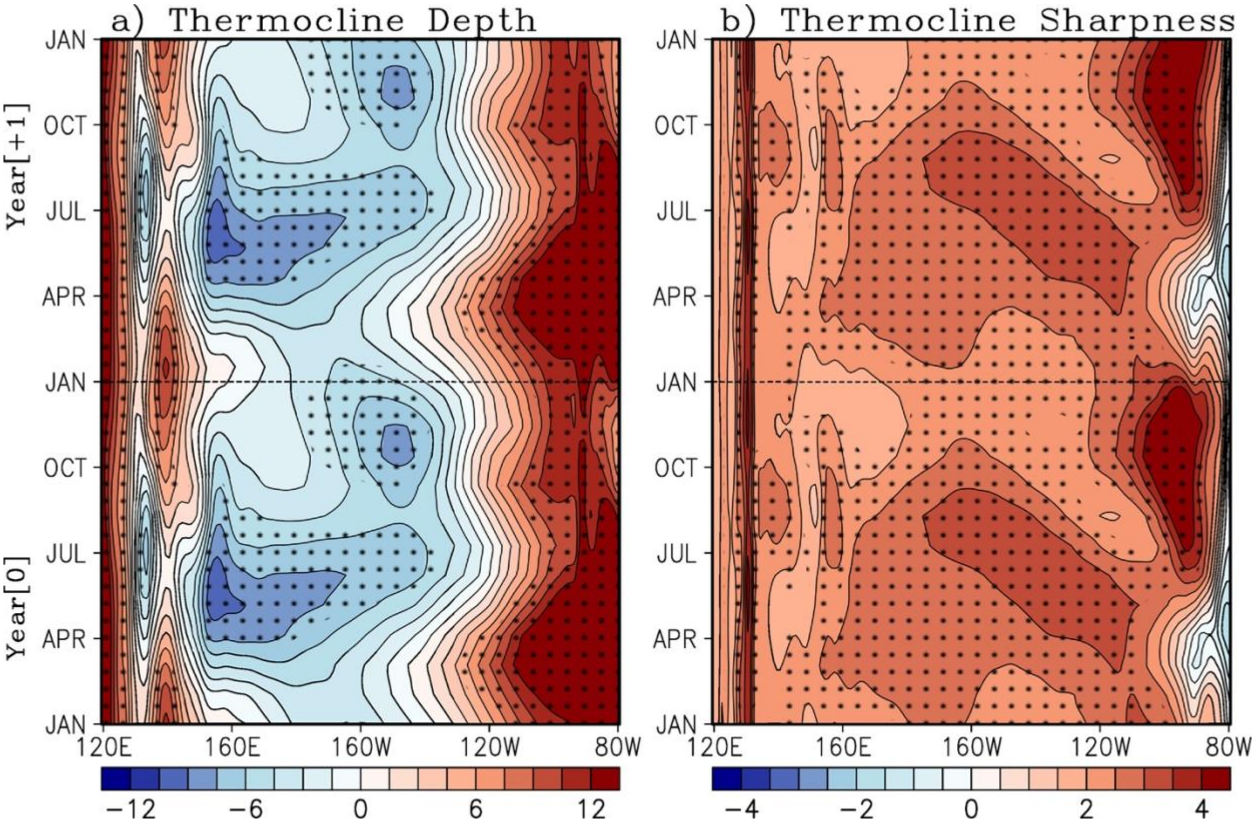

Supplementary Figure 8. CESM-LENS two-year repeated monthly climatology of the differences in the 21st minus the 20th Century mean states of a) thermocline depth [m] and b) intensity of the thermocline (i.e., sharpness) as measured by $\frac{\partial T}{\partial z}$ at the thermocline depth [10^{-2} °C/m]. Hatching indicates statistical significance at the 95% confidence level based on a bootstrapping technique (see Methods).

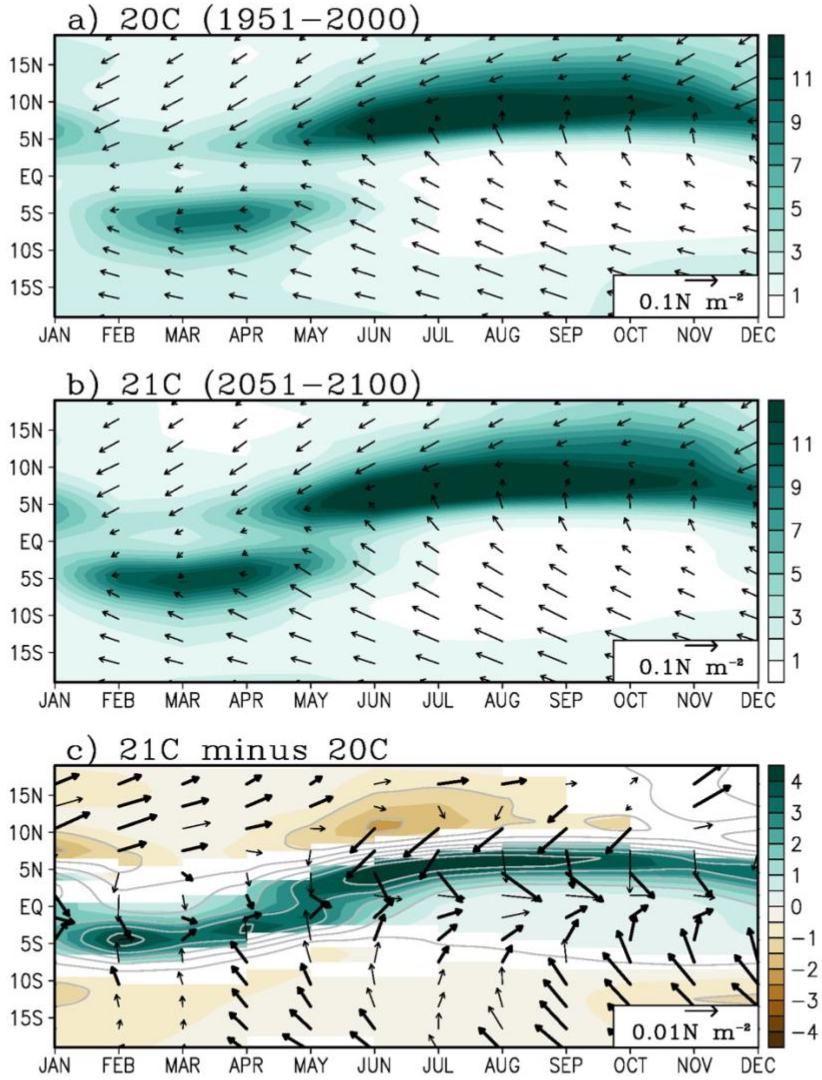

Supplementary Figure 9. Latitude-time precipitation (color, mm day^{-1}) and wind stress (vector, N m^{-2}) climatology from CESM-LENS zonally averaged from 140°W-80°W for the a) 20C period (1951-2000), b) 21C period (2051-2100), and c) the difference between the 21C minus 20C periods. Precipitation and wind stress vectors differences in panel c) that are significant at a 95% confidence level using a bootstrapping subsampling technique are shown by shading color and thick vectors respectively.

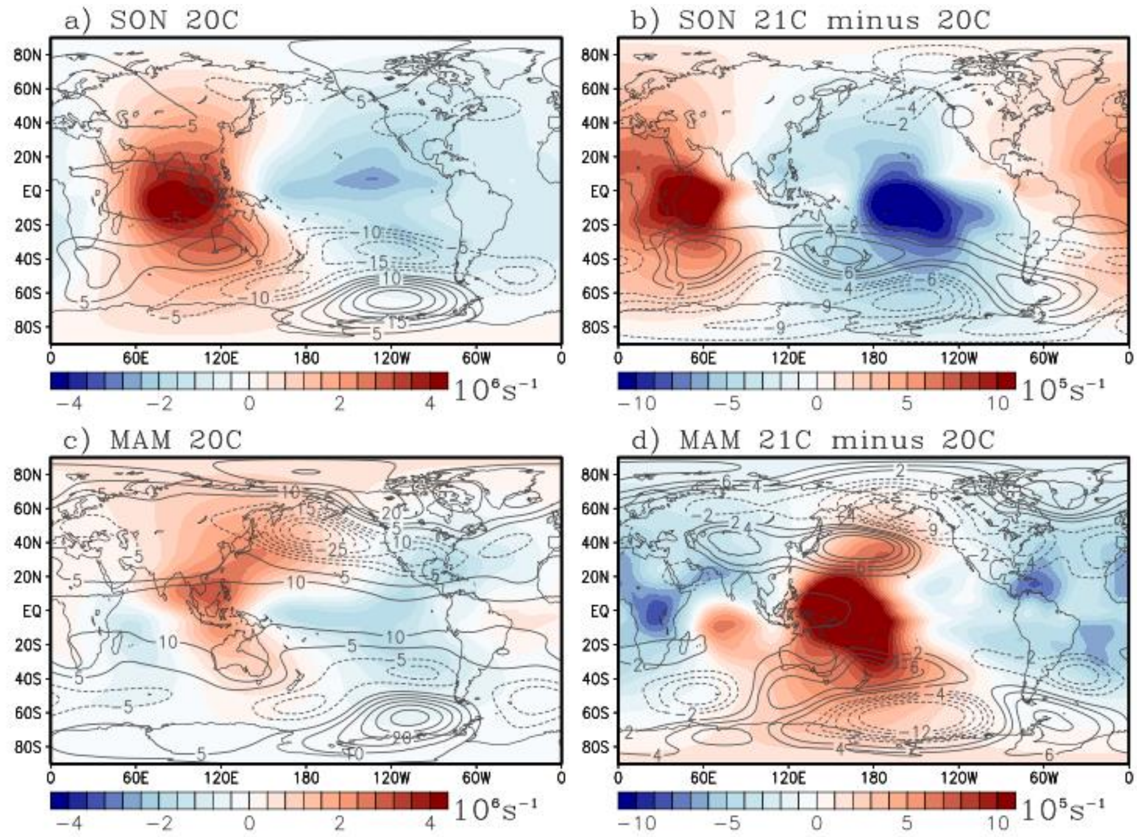

Supplementary Figure 10. a) Composite analysis of 200hPa velocity potential (color, interval 10^6 s^{-1}) and 500hPa geopotential height (contour, interval 5 m) anomalies during El Niño events for September-October-November (SON, or developing year-0) for late 20th Century and b) the projected changes (i.e., 21C minus 20C) of 200hPa velocity potential (color, interval 10^5 s^{-1}) and 500hPa geopotential height (contour, interval 2 m). Panels c) and d) are similar to a) and b) respectively but for the March-April-May (MAM, or decay year+1). See Methods for Definition of El Niño.

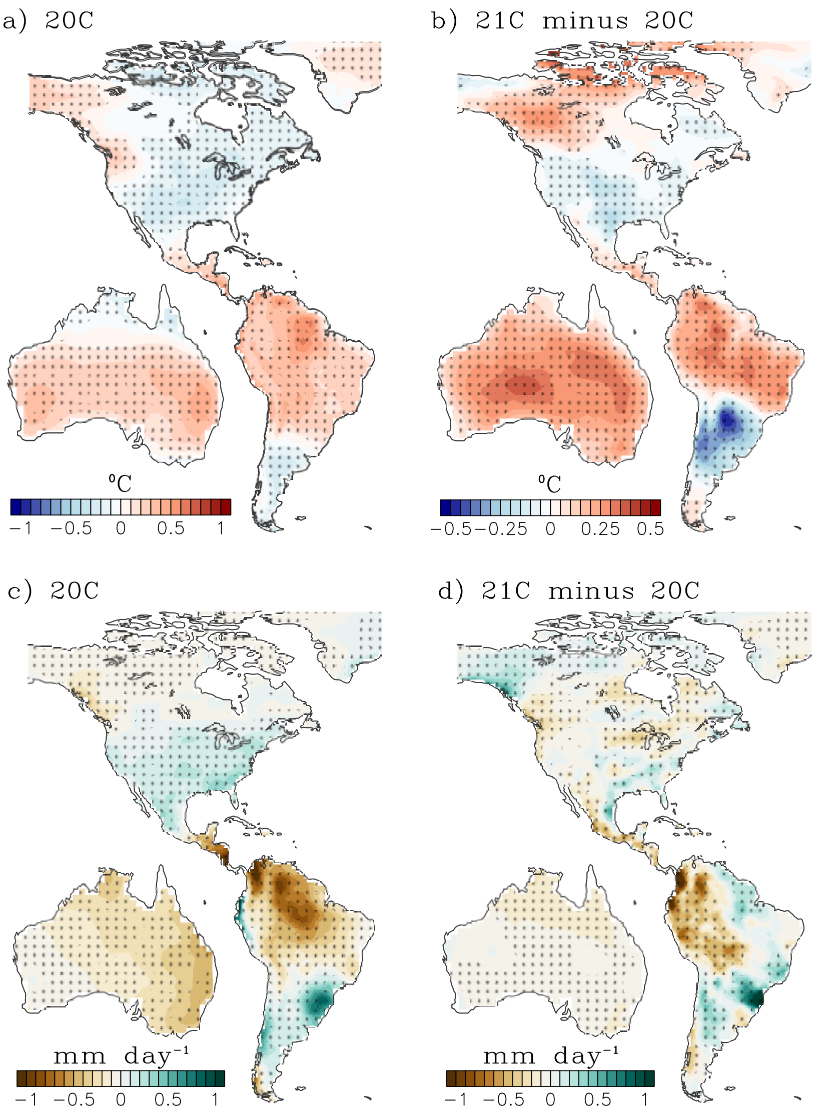

Supplementary Figure 11. Remote effects of El Niño event on surface temperature [blue-red, °C] and precipitation [brown-green, mm day^{-1}] and their projected changes from CMIP6. a) Surface temperature and c) precipitation composite during September-October-November (SON, or growth year-0) for late 20th Century El Niño events (see Methods for Definition of El Niño). Projected changes in b) temperature and d) precipitation composite (21C minus 20C) during SON (or growth year-0). Stipples indicate anomalies that are significant at the 95% level based on a bootstrapping technique (see Methods).

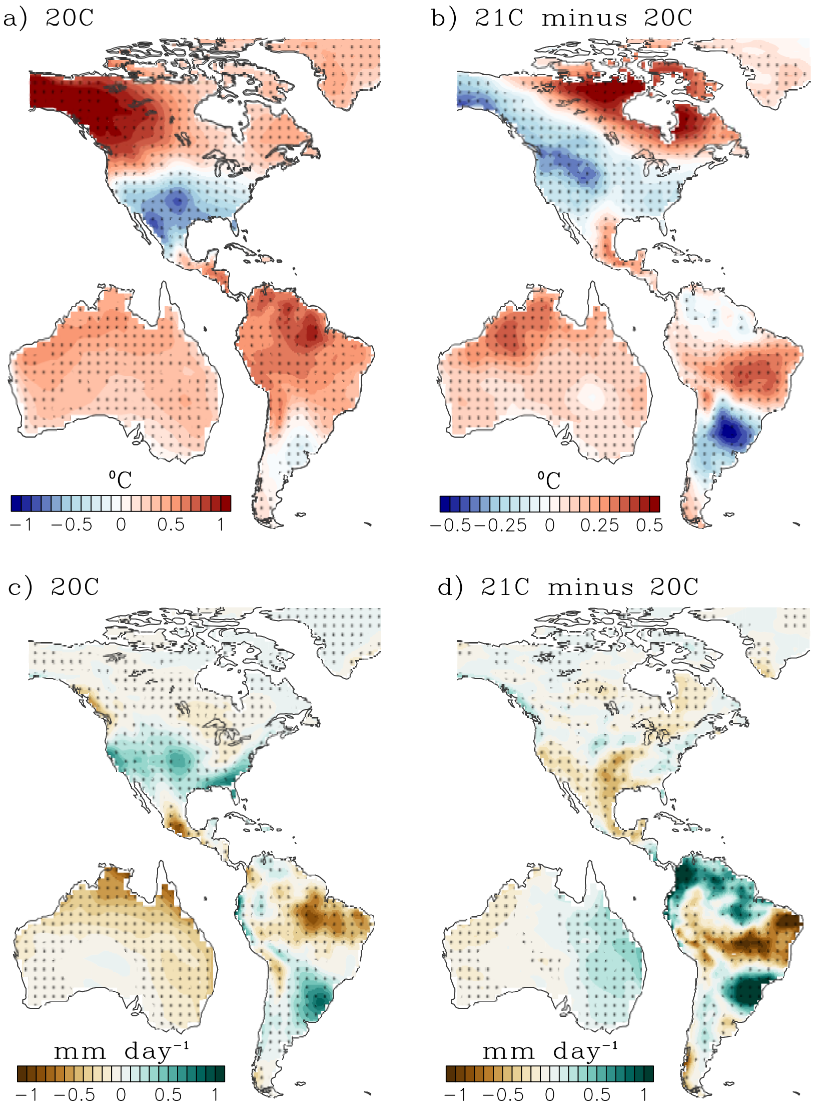

Supplementary Figure 12. As in supplementary Fig. 11, but for March-April-May (MAM, or decay year+1).

Supplementary Note 7: Analysis of the spatio-temporal evolution of El Niño in CMIP6 and CESM-LENS

Please note that the CMIP6 models chosen here are those that have availability of historical and future projections under the SSP585 scenario (at the moment of this writing). Also note that we choose the SSP585 scenario as this is the one that more closely relates to the CMIP5-RCP8.5 scenario originally employed for the CESM-LENS.

Supplementary Figure 13 shows, similar to Fig. 1 in the paper, the spatio-temporal evolution of SSTA during 20C El Niño events for the observed, CESM-LENS, and several CMIP6 models. To compare the models in a more quantitative way, we use a Taylor diagram, as shown in Supplementary Fig. 14. The Taylor diagram relies on statistical quantities that are closely related among each other as described in eq. S1 below. Here, *obs* (*model*) is the spatio-temporal evolution

of observed (modeled) SSTA as in Supplementary Fig. 13. *RMSE* is the root mean square error between the observed and modeled SSTA, σ_{obs} *(*σ_{model}*) is the observed (modeled) standard deviation, and cor* is the anomaly correlation between observed and modeled SSTA.

$$[RMSE(obs, model)]^2 = \sigma_{obs}^2 + \sigma_{model}^2 - 2\sigma_{obs}\sigma_{model}cor(obs, model) \qquad eq. S1$$

From Supplementary Fig. 14 it is observed that the CESM-LENS model, has a high spatio-temporal correlation (*cor=0.9)* and an *RMSE=0.52σ* with respect to the observed SSTA evolution during El Niño. The ability of CESM-LENS in reproducing the observed evolution of El Niño is relatively average when compared to supposedly more up-to-date CIMP6 models. Among the CMIP6 models, the GFDL-CM4, GFDL-ESM4, HadGEM3, and CESM2 models show the most accurate representation of the spatio-temporal evolution of SSTA during El Niño events. However, all models shown here show a relatively small RMSE when compared to the error saturation level (i.e., $RMSE > \sqrt{2}\sigma$).

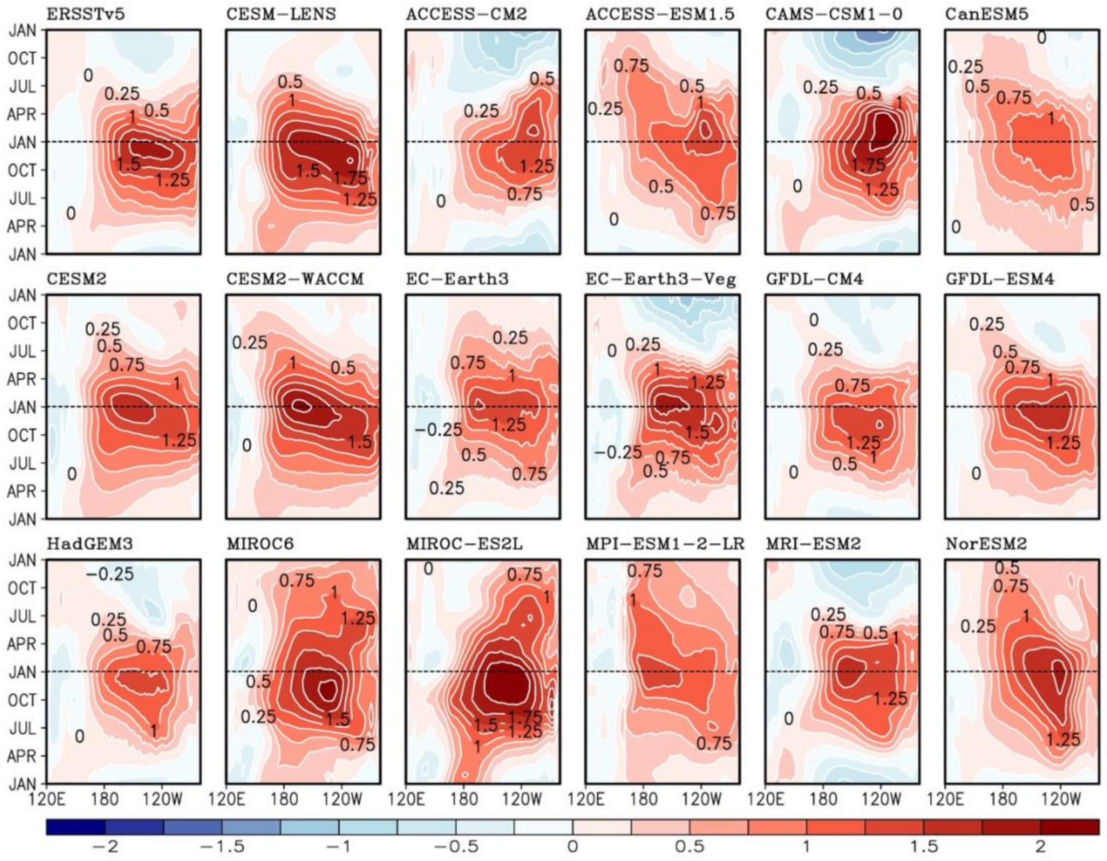

Supplementary Figure 13. Longitude-time evolution of observed and simulated El Niño events. Composite of monthly equatorial Pacific sea surface temperature anomalies [SSTAs, °C, averaged 5°S–5°N] for El Niño events for the 20C period (1951-2000) from the observational reconstruction of ERSSTv5 (top-left) and simulations from CESM-LENS and the sixteen CMIP6 models. Ordinate represents time from January of the onset year to December of the decay year.

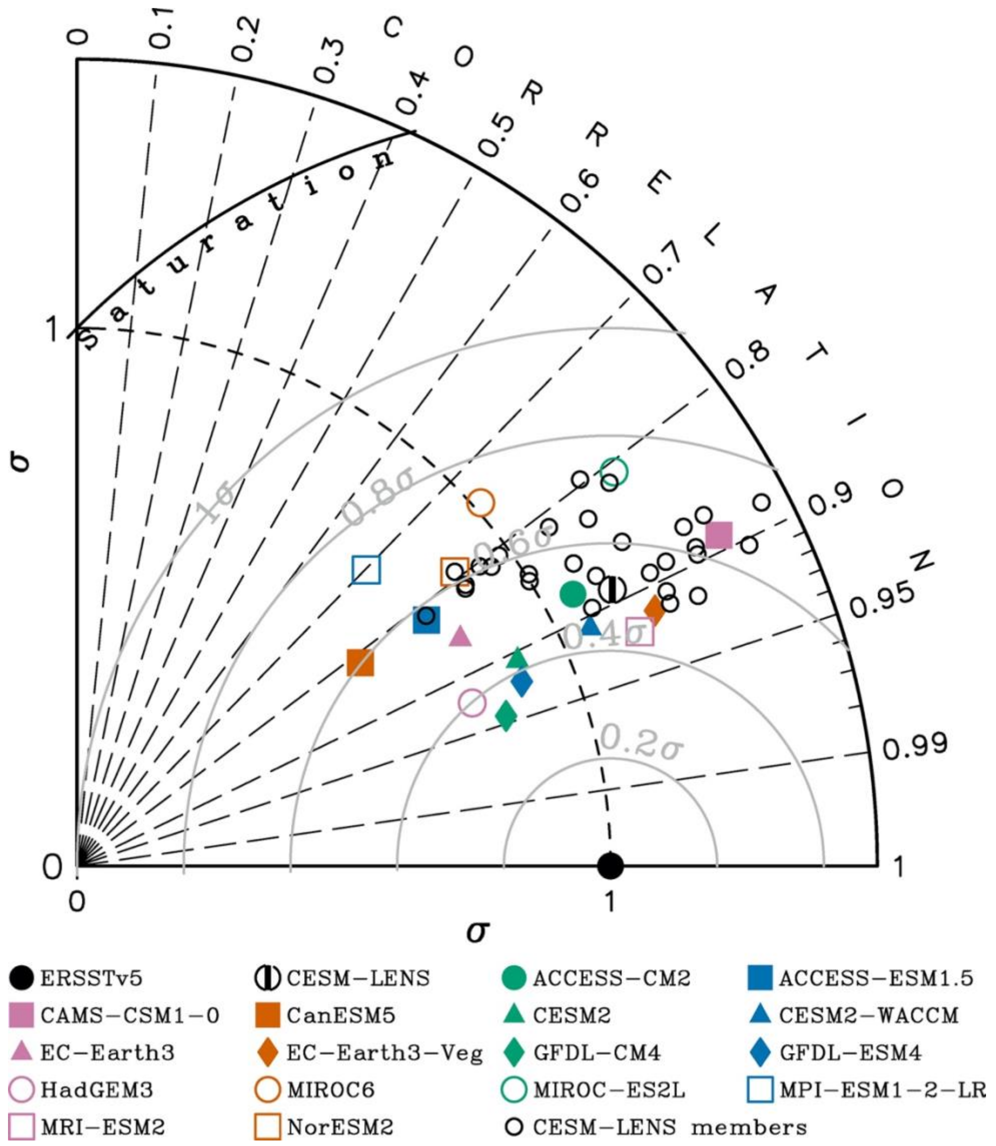

Supplementary Figure 14. Quality assessment of several CMIP6 models and the CESM-LENS model in reproducing the observed spatio-temporal evolution of SSTA during 20C El Niño events. Taylor diagram compares the standard deviation (radial axis) and spatiotemporal anomaly correlation (azimuthal axis) between the observed (i.e., ERSSTv5) and modeled SSTA evolution shown in supplementary Fig. 13. The concentric semicircles centered at ERSSTv5 represent the root mean squared error (RMSE) in standard deviation units (σ). Note that the RMSE and σ are all standardized based on the ERSSTv5 standard deviation, to facilitate comparison among different models. Each individual ensemble member from CESM-LENS is shown by open-black circle.

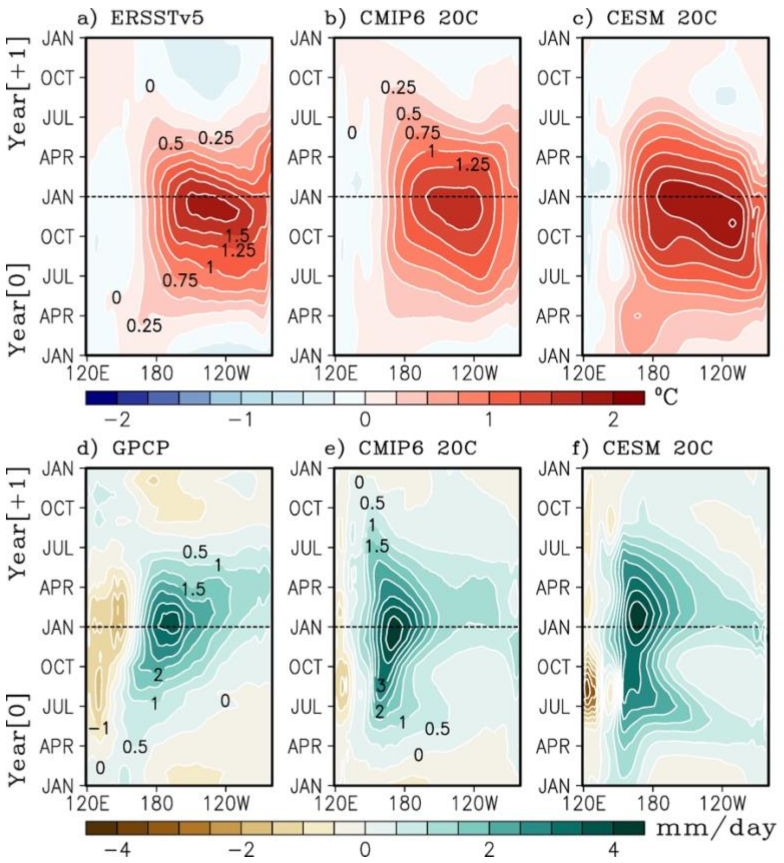

Supplementary Figure 15. Spatio-temporal evolution of equatorial Pacific SSTA and precipitation anomaly during El Niño events. a) Composite of observed SSTA [°C] from ERSSTv5 and d) observed precipitation anomalies [mm day^{-1}] from GPCP averaged 5°S–5°N for El Niño events for the period of 1979-2000). Panels b) and e) show the composites for SSTA and precipitation anomaly from the CMIP6 ensemble mean for the 20C period (1951-2000). Similarly, c) and f) show the composites for the CESM-LENS ensemble mean for the 1951-2000 period. Ordinate represents time from January of the onset year (Year 0) to December of the decay year (Year 1).

Supplementary Note 8: Interannual anomaly definition:

For CESM-LENS (Method#1, here forward), the ensemble mean is subtracted for each ensemble member, then a monthly climatology is further removed from the resultant anomalies. This is done to avoid the potential for each ensemble member having a slightly different climatology. This method, should take care of any trend (including non-linear) due to external forcing as well as CESM model bias.

For CMIP6 (Method#2, here forward), each model has its own physics and thus bias, so the ensemble mean approach is not applicable. To compute anomalies in CMIP6, we remove a 30-year running mean climatology, similar to what is done at NOAA/CPC to identify ENSO events.

Supplementary Fig. 16 (panel a) shows the Niño3.4 SSTA timeseries reconstructed using the two method for a randomly selected ensemble member. Supplementary Fig. 16 (panel b) shows a Taylor diagram from all 30 CESM-LENS ensembles computed using both methodologies outlined

above. Note that the temporal correlation between the two methods is higher than 0.95 for all ensembles, with root mean square errors smaller than 35 percent of the standard deviation of Niño3.4 (RMSE < 0.35σ). The two methods yield nearly identical results. However, only Method#2 is appropriate for use with multi-ensemble simulations of different models (e.g., CMIP6), while both methods are appropriate for multi-ensemble simulations of a single model (e.g., CESM-LENS). Method#1 was chosen for CESM-LENS as it is significantly less computational demanding, especially when quantifying multi-dimensional anomalies (e.g., sub-surface temperature and velocities) as a function of latitude, longitude, depth, time, and ensemble member.

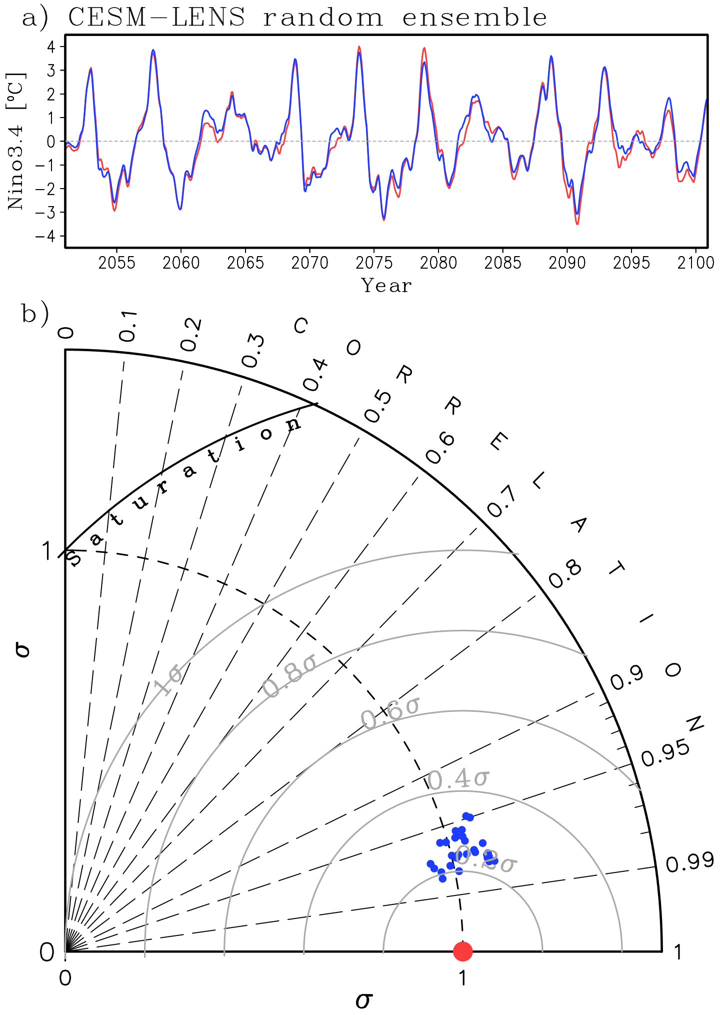

Supplementary Figure 16. a) Niño3.4 SSTA timeseries for a randomly chosen ensemble member of the CESM-LENS computed from the deviation from the ensemble mean (i.e., Method#1, red) and from the deviation from a 30-year running average (i.e., Method#2, blue). b) Taylor diagram depicting the standard deviation (abscissa), correlation (azimuth), and root mean square error (concentric circles) of the two methods of defining Niño3.4 SSTA anomalies for all 30 ensembles from the CESM-LENS. All timeseries were normalized based on the standard deviation from Method#1 (red dot) to facilitate comparison against Method#2 (blue dots).
